# Development and validation of the Chinese Quality of Life Instrument

**DOI:** 10.1186/1477-7525-3-26

**Published:** 2005-04-16

**Authors:** Kwok-fai Leung, Feng-bin Liu, Li Zhao, Ji-qian Fang, Kelvin Chan, Li-zhu Lin

**Affiliations:** 1 Department of Occupational Therapy, Queen Elizabeth Hospital, Kowloon, Hong Kong SAR, China; 2 The First Affiliation Hospital, Guangzhou University of Traditional Chinese Medicine, Guangzhou, China; 3 Research & Development Division, School of Chinese Medicine, Hong Kong Baptist University, Hong Kong SAR, China; 4 School of Public Health, Sun Yat-Sen University, Guangzhou, China

**Keywords:** Quality of life, self-reported health status, theory driven approach, Ying-yang, structure fitness, validation, psychometric properties, the Chinese Quality of Life instrument

## Abstract

**Background:**

This paper describes the development of the Chinese Quality of Life Instrument (ChQOL) which is a self-report health status instrument. Chinese Medicine relies very much on asking subjective feelings of patients in the process of diagnosis and monitoring of treatment. For thousands of years, Chinese Medicine practitioners have accumulated a good wealth of experiences in asking questions about health of their patients based on the concept of health in Chinese Medicine. These experiences were then transformed into questions for the ChQOL. It is believed that ChQOL can contribute to the existing Patient Report Outcome measures. This paper outlines the concept of health and disease in Traditional Chinese Medicine, the building of the conceptual framework of the ChQOL, the steps of drafting, selecting and validating the items, and the psychometric properties of the ChQOL.

**Methods:**

The development of the ChQOL was based on the concept of health in Traditional Chinese Medicine with a theory driven approach. Based on the results of literature review, the research team developed an initial model of health which encompassed the concept of health in TCM. An expert panel was then invited to comment and give suggestions for improvement of the initial model. According to their suggestions, the model was refined and a set of initial items for the ChQOL was drafted. The refined model, together with the key domains, facets and initial items of the ChQOL were then mailed to a sample of about 100 Chinese medicine practitioners throughout Mainland China for their comments and advice. A revised set of items were developed for linguistic testing by a convenience sample consisting of both healthy people and people who attended Chinese Medicine treatment. After that, an item pool was developed for field-testing. Field test was conducted on a convenience sample of healthy and patient subjects to determine the construct validity and psychometric properties of the ChQOL.

**Results:**

Construct validity was established by various methods, i.e. the internal consistency in all facets and domains were good; the correlation between facets to domain, and domains to overall ChQOL correlation were high; confirmatory factor analysis showed that the structure fitness of all facets, domain and overall structure were good with CFI > 0.9. Test-retest reliability was also good, especially in the domain scores with ICC value ranging from 0.83 to 0.90. No ceiling or floor effect was noted which indicated that ChQOL can be applied to subjects with a wide range of health status. Most facet scores, domain scores and the overall CHQOL scores were able to discriminate groups of subjects with known differences in health status. The ChQOL had mild positive convergence with the other generic health related QOL measures, i.e. the WHOQOL-100 and the SF-36, with moderate correlations.

**Conclusion:**

In conclusion, the study indicated that the ChQOL is conceptually valid with satisfactory psychometric properties. It can provide additional information on health and QOL on top of the existing generic health related QOL measures. Furthermore, it forms basis for further testing and applications in clinical trials.

## Background

Traditional Chinese Medicine (TCM) has been practiced in China for thousands of years. Throughout history, millions and millions of Chinese people have been cured by various forms of TCM, including the use of Chinese herbal medicine, acupuncture, and Qigong, etc. In the recent few decades, the use of Chinese medicine (CM) is becoming more popular on its own, and also as a kind of complementary treatment to Western Medicine throughout the world [1]. In the West, much attention is paid on the use of CM in the alleviation of painful symptoms and undesirable side effects of radical pharmaceutical and radiological treatments.

TCM practitioners do not rely on biomedical and radiological examinations for their diagnosis and treatment of patients. (Modern CM practitioners may take biomedical and radiological examinations as reference) They rely heavily on observable signs and subjective feelings of patients. The four fundamental techniques that have been used by CM practitioners for thousands of years for diagnosis and treatment are: observation (looking for visible signs), auscultation and olfaction (listening and smelling), interrogation (asking questions), and palpation (felling the pulse) [2,3]. Throughout the centuries, CM practitioners have accumulated valuable experiences on capturing very detailed and accurate information about the signs, symptoms and feelings of their patients. We believed that these experiences may be useful in applying in health related quality of life (HRQOL) measures and other forms of patient reported outcome measures.

Traditionally, Western Medicine and Chinese Medicine are using very different ways in testing their clinical efficacy. Randomized clinical trial (RCT) technique is a key method adopted in Western Medicine. In Chinese Medicine, clinical efficacies are observed, categorized and recorded descriptively in the literature. In the past fifty years, many scholars in Mainland China and in the Western countries in North America, Europe and Australia, etc, have been studying CM with RCT methods. The scientific study on the efficacy of CM using RCT method is becoming more and more popular. QOL indicators were used as secondary outcome indicators in many of these studies. However, the efficacies of CM on QOL were not particularly evident in these studies. There is a general impression among CM practitioners that the currently available QOL instruments may not be sensitive enough to detect the health changes that is regarded as important in CM. For examples, in CM, appetite and digestion, routine of urination and bowel, facial and lips colour, spirit in the eyes, and adaptation to climates and seasons are very important indicators of health status. However, these indicators are usually not included in common HRQOL measures.

A group of CM practitioners and QOL researchers in Hong Kong and Mainland China came together and worked towards a mission of developing a HRQOL instrument, the Chinese Quality of Life Instrument (ChQOL), which embedded the concept of health in TCM. We believed that the experience of CM might further enhance the advancement of quality of life assessment and research. We also believed that a HRQOL instrument that was developed basing on the concept of health in TCM might be more sensitive to the efficacy of CM. Besides adopting a theory driven approach, we had applied a set of standardized procedures that was commonly recognized and accepted in Western Medicine in the development of the ChQOL [4].

## Method

### Establish the initial ChQOL structure

Based on the results of literature review on the concept of health and disease in TCM, the research team developed an initial model of the ChQOL which encompassed the concept of health in TCM. The model consisted of the essential domain of HRQOL. Then, an expert panel consisted of several CM scholars from different fields of practice in Chinese medicine were formed. These experts came from various areas of Chinese Medicine, including clinical experts, pharmaceutical experts and academics who study theories of TCM. A half-day expert panel meeting was held. The panel was asked to comment on the initial domain model, and to proposed strategies on developing facets under the domains. The results of the penal meeting were used to produce the domain and facet structure that guided the drafting of items.

### Draft items

Items of the ChQOL were supposed to be indicators of health in TCM. They were written in wordings that were commonly used in the communication between CM practitioners and their patients in soliciting clinical information. The items were phrased in form of questions asking about intensity, frequency, capacity or satisfaction of signs, symptoms, and feelings where appropriate. Subjects could rate the items with a set of five-point ordinal scales with descriptors. For each of the facet, 4 to 8 items would be drafted.

### Review of the drafted items by clinician

The drafted items, the domain and facet structure of the ChQOL, were then mailed to a convenience sample of about 100 CM practitioners throughout Mainland China for their comments and advice. They were asked to give their comments in a standard reply form indicating if they agreed on the domains, facets and the items. They were asked to give suggestions to improve the items and to add new items.

With the input from these CM practitioners, a revised set of items were developed for the cognitive debriefing step. A small convenience sample consisted of both healthy people and patients, who were receiving CM treatment, were recruited. They were asked to comment on the linguistic and semantic clarity of the items. They were also asked to suggest improvement in the wordings of the items. After that, an item pool was developed for field-testing.

### Select response scales

The 5-points response scales in the Chinese version WHOQOL-100 were adopted in the ChQOL. There were several types of response scales that suit intensity, frequency, capacity, and satisfaction questions. The scales were developed in a way that the 3 descriptors between the two anchor points lie approximately at the 25%, 50% and 75% of the scale. The WHOQL-100 responses scales were assumed to be interval scale for data analysis [5,6].

### Facet and domain scores

The response scales ranged from 1 to 5. In most items, score 1 referred to the lowest QOL and 5 referred to the highest QOL. In some items, reverse of polarity might be required before formulating the facet scores.

In formulating the facet scores, two methods were tested. The first method assumed that the weights of the items in the same facet were the same. Raw facet score was derived by summing the item scores in the facet and then transformed to a 0 -100 facet scale. The same way was used to derive the domain scores. In the second method, the coefficients between the items and the corresponding facets, obtained from the structural equations, as the weights for the items. Raw facet scores were derived by summing up the product of individual item score and the corresponding coefficient. The raw facet score was transformed to a 0 -100 facet scale. The same ways were used to derive the domain scores.

### Field test

A convenience sample of healthy and patient subjects was recruited to test the domain and facet structure and to examine psychometric properties of the ChQOL. In the field test, all the subjects were asked to fill in a set of questionnaire which consisted of the items in field test version of the ChQOL, the Chinese version SF-36 [7,8], and the Chinese version WHOQOL-100 [7,9]. The subjects were asked to comment on their own health status by answering a single question on self-perceived health status, i.e. rate their health status as "very bad" or "bad" or "neither good nor bad" or "good" or 'very good" Further, they were also asked to comment on their illness condition by responding "having no illness" or "having a stable chronic illness" or "having an acute illness". Subjects were asked to provide personal particulars as well. Assistances were provided to those who had difficulties in reading and writing due to various reasons.

### Build the final ChQOL structure

Exploratory factor analyses using Principle component extraction method with Varimax rotation and Eigenvalue >1, were used to examine the items in each of the domains. The purpose was to select items that best represent the facets, and to reduce items that did not fit in well with other items in any facets under the domain. Confirmatory factor analyses were then conducted to confirm the item-facet and facet-domain structure of the domains. Comparative fit index and the Chi square value were reported to show the structure fitness. The facet scores were used to test the overall facet-domain structure of the ChQOL.

### Examine psychometric properties of the CHQOL

A series of analysis were conducted to test the psychometric properties of the ChQOL. Distributions of the facets and domain scores were examined and reported in terms of range, means, and standard deviation. The existence of ceiling and floor effect were also tested. Correlation between facet and domain scores, and inter-domain scores were examined. Pearson correlation coefficients were reported. Internal consistency of the facets was tested by examining the Cronbach's alpha value. Test-retest reliability of the facet and the domain scores were examined by a sub-sample within 2 days and the ICC(1,1) values were reported. Convergent validity was established by correlating the domain scores of the ChQOL with the 6 domain scores of the WHOQOL-100 and the 8 facet scores of the SF-36. ANOVA tests were used to examine the discrimination validity of the ChQOL. It was performed by checking if the domain scores of the ChQOL can discriminate the self-perceived health status, and the illness status of the subjects.

### Study conceptual overlapping with the WHOQOL-100 and SF-36

Exploratory factor analyses were conducted with the facet scores of the ChQOL and two commonly used generic HRQOL measures separately. The two measures were the Chinese version WHOQOL-100, and the Chinese version SF-36. The purpose was to examine if there were overlapping of the facets at the domain level in the three measures.

### Statistical software

SPSS version 11.0 was used in data analysis [10]. The software EQS version 6.5 b was used in examining structure fitness of various structure models. [11].

## Results

### Concepts of Health in TCM

The first task of the study was to explore the theoretical bases for the ChQOL by reviewing both ancient and modern CM literatures. We could not find an established theory on "concept of health in Chinese Medicine" in the TCM literature. Health state is being described in various TCM theories in different contexts. Our conceptual model for the ChQOL had to be deducted from our understanding of the core concepts of TCM.

The fundamental theoretical basis of TCM originates from the ancient Chinese philosophy of Yin-yang [2,3]. The theory of Yin-yang can be understood as a conceptual tool for man to understand the order, principles and relationship of the universe, nature, objects and every day life phenomena. The nature, objects and day-to-day phenomena can be classified into two opposite but complementary categories, which are understood from two opposite perspectives, i.e. Yin and Yang. Although Yin and Yang are opposite to each other, they are mutually dependent and mutually transformable. Further, Yin and Yang are mutually restrictive and interactive, and in equilibrium with each other. Examples in nature are: earth to sky, moon to sun, water to fire, coldness to warmth, descending to ascending, static to motion, material to function, etc. Examples of Yin-yang in human are: woman to man, body organ to body function, physical form to spirit, etc. Examples in disease states are: exterior to interior syndrome, cold to heat syndrome and deficiency to excessive syndrome, etc.

Under the theory of Yin-yang, health is a state of harmonization of the "Physical Form" and "Spirit" (), harmonization of "Man" and "Society" (), and harmonization of "Man" and "Nature" (or environment) () [12].

Physical form (Xing, ) refers to the bodily structure of the human beings. Spirit (Shen, ) has many meanings. It can also refer to the general outward manifestations of the life activities and the mental activities, which include consciousness and reasoning of human mind. It can also refer to the functional manifestations of the changes in the body and their intrinsic laws. In accordance with CM, the physical form of the body is the bases for producing the spirit, while the spirit can regulate and control the body. They depend on each other for their existence, and the unity of the body and spirit is the main assurance of one's survival [2].

The concept of the Seven-Emotion refers to various human emotions. It can be regarded as emotional consequences of the interactions between an individual with the external environment, including both human and natural environment. It can also be the manifestation of the internal health status of an individual. According to this theory, human being has 7 basic emotions, which are: joy (), anger(), worry(), pensiveness(), grief(), fear(), and anxiety(). The 7-emotions theory stresses that emotion and body are linked and mutually affected. Therefore, emotions can be the determinants of health as well as the indicators of health in a different context [2,13]. In TCM literature, the relationship between emotion and health was elaborated in details. Liver relates to anger, heart to joy, spleen to pensiveness, lung to worry, and kidney relates to fear. Emotion disturbances due to stress coming from outside of the body affect the corresponding viscera organs and results in illnesses. For example, anger impairs liver and over-joy impairs heart. Worry leads to abnormality of lung and spleen. Pensiveness affects heart and spleen. Grief causes problems in heart and lung. Fear gives rise to diseases of heart, kidney and liver. Fright affects heart and liver. In fact, worry, pensiveness, fright and fear usually appear at the same time and causing multi-organs or systemic diseases.

### Concepts of diseases in TCM

In the TCM literature, there are much more description on diseases rather than health. Disease state is the result of breaking down of the equilibrium of Yin and Yang. In the disease state, there is an excess or deficiency of either Yin or Yang. In describing a disease, two major theories are used. They are the 8-Principle Syndrome Differentiation Theory () and the Visceral Syndrome Differentiation Theory () [2]. These two theories can be viewed as clinical reasoning tools for classifying specific health states and syndromes, and for diagnosing illnesses. The 8-Principle Syndrome Differentiation Theory classifies syndromes by using 4 different dimensions, which are: Yin or Yang syndrome (), exterior or interior syndrome (), cold or heat syndrome () and deficiency or excessive syndrome (). The body organs or body functions that are affected by these syndromes are classified by the Visceral Syndrome Differentiation Theory. By using these two classification systems on various syndromes, a diagnosis can be derived, and treatment can be planned accordingly.

Treatment in CM is a process of regulating and re-establishing the balance of Yin and Yang through reducing the redundancy of Yin or Yang () and reinforcing the deficiency of Yin or Yang () [3]. Herbal medicine, acupuncture, and Qi Gong are used as major treatment modalities in CM.

### Theoretical framework of the ChQOL

In this study, we adopted the concept of health rather than the concept of disease in TCM as the theoretical base of the ChQOL. The concept of positive health was used to form the domain-facet structure of the ChQOL. The research team did not use the 8-Principal Syndrome Differentiation Theory and the Visceral Syndrome Differentiation Theory to construct the initial structure of the ChQOL. These 2 theories might be good for developing disease specific modules since they elaborate more on the diseases rather than health.

**Figure 1:**
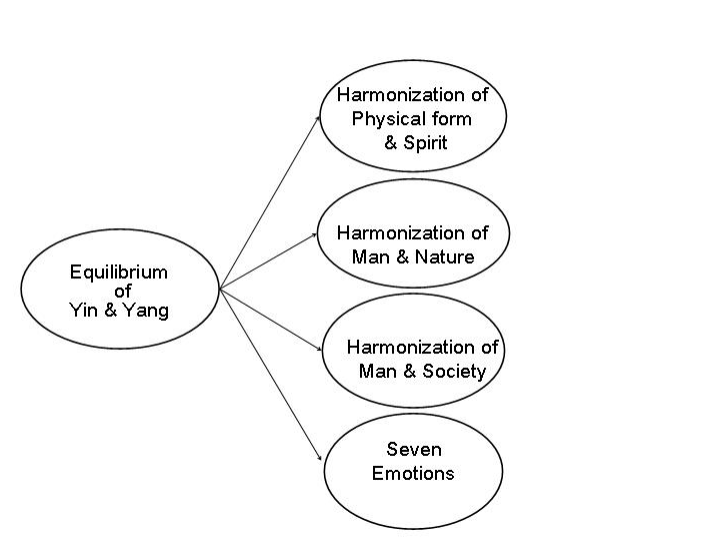
Initial structure model of the ChQOL for the purpose of drafting of items

The research team had proposed an initial structure model for the ChQOL with 4-domains (Figure 1). The four domains were: the harmonization between Physical Form and Spirit, harmonization between Man and Society, harmonization between Man and Nature, and the Emotions. Theory on harmonization of Physical Form and Spirit was the core domain because it is much richer in its coverage and content. Since the conceptual relationship of the domains was not clear, several possible models could be developed. At the initial stage, the four domains were arranged in parallel with each other.

An expert penal consisted of 5 CM experts were invited to comment on the initial model. There were two CM clinicians, one CM pharmaceutical expert, and one academic who was an expert in TCM theory. A half day panel meeting was conducted. In the meeting, the penal members supported the initial model in principle. The penal opined that the domain on the harmonization of Physical Form and Sprit could be viewed as two related but separate domains. It was because of the fact that quite a number of facets could be proposed under each of these two domains; and the two domains could provide some unique information about the general health of an individual.

Based on the discussion in the penal meeting, the research team had revised the domain structure of the ChQOL. The original domain on Physical Form and Spirit was separated into two domains. Therefore, the revised model had 5 domains. In TCM literature, there was no clear description of the hierarchical relationship of various health concepts and body functions, i.e. there were no clear classification of domain and facets for health. The team had proposed facets for the domains basing on their interpretation of TCM literature and their clinical experiences. These facets were important indicators for reflecting the equilibrium of Yin and Yang in the respective domains.

In the Physical Form domain, 8 facets were proposed, which were: body build, complexion, sleep, appetite and digestion, stamina, breathing, sex, bowel and urination. In the Spirit domain, 4 facets were proposed, which were: thinking, verbal expression, consciousness, and spirit of the eyes. In the Emotion domain, 5 facets were proposed, which were: joy, anger, worry & pensiveness, grief, fear & anxiety. No facets were developed under the domain on harmonization of Man and Society, and harmonization of Man and nature.

### Item pool

Based on the revised domain and facets, we started to draft items for the ChQOL. The team was successful in drafting many items for the Physical Form domain, Spirit domain and Emotion domain. The numbers of items drafted were 33, 13, and 23 in the Physical Form, Sprit, and Emotion domain respectively (Table 1).

**Table 1:** Number of items under the facets in various stage of development

	**Number of items**			
	
**Domains and facets**	**Initial draft by the research team**	**After review by 76 TCM practitioners**	**After cognitive debriefing**	**After factor analysis**
Physical Form Domain	33	37	36	20
Body build	7	7	7	0
Complexion	3	5	5	4
Sleep	4	4	4	3
Stamina	3	4	4	6
Appetite & digestion	6	7	7	4
Breathing	1	2	1	0
Sex	1	2	2	0
Bowel & urine	4	2	2	0
Adaptation to climate	4	4	4	3

Spirit Domain	13	16	16	12
Consciousness	3	5	4	3
Thinking	5	4	5	5
Spirit of the eye	2	3	3	2
Verbal expression	3	4	4	2

Emotion Domain	23	25	26	18
Joy	4	5	6	4
Anger	4	5	5	5
Worry & Pensiveness	7	5	5	0
Grief	4	5	5	0
Fear & Anxiety	4	5	5	3
Depressed mood	0	0	0	6

Total:	69	78	78	50

However, in the process of item drafting, it was found that CM practitioners did not ask many questions related to the domain on Harmonization between Man and Society, This might be due to the fact that CM practitioners focus more on health and diseases rather than social relationship in their day-to-day clinical practice. The research team managed to draft some questions on this domain. However, the items were not usual asked by CM practitioner in their practice; and further, these items were very much similar to the items in many existing QOL instruments, which did not show much connection to CM. Therefore, the team decided not to use these items because this might deviated from the original intention of using the genuine knowledge and experiences of TCM in developing the ChQOL.

For the domain on the harmonization between Man and Nature, only 4 items related to adaptation to seasons and climate were drafted. These items could form a domain. However, in CM, adaptation to seasons and climate could also be regarded as a facet under the Physical Form domain. Therefore, the research team decided to re-group these items as a facet under the Physical Form domain so that the overall ChQOL structure could be simplified. This facet was named as adaptation to climate. As a result, there were 9 facets under the Physical Form domain.

The conceptual structure of ChQOL was then revised as a three domains structure, which were the Physical Form domain, the Spirit domain, and the Emotion domain, with a total of 69 items (Table 1). By dropping the domain on Harmonization of Man and Society and Harmonization between Man and Nature, the coverage of ChQOL became narrower and focused more on health status rather than a broader sense of quality of life.

### Clinician review on the ChQOL structure and the drafted items

The refined domains and facets model and the 69 items of the ChQOL, were then mailed to a sample of about 100 Chinese Medicine practitioners for their comments and advice. 76 sets of comment were received in a standard reply form. Nearly all members in the group agreed on the domains and facets structure of the ChQOL. Some of them expressed reservations but could not make concert suggestions. With their input, the domains and facets structure of the ChQOL were further confirmed. Many of the CM practitioners made suggestions on refining certain items. Some pointed out that some items might have different interpretations in different people, some were confusing, some asked two things in one question, etc. The team reviewed each suggestion and many of them were adopted to revise the original items. Some additional questions for the facets were suggested. All items that could expand the scope of coverage for the facets were adopted. As a result, a total of 78 items were then developed for the cognitive debriefing step.

### Cognitive Debriefing

Cognitive Debriefing was performed with a convenience sample of 15 adult subjects that included both healthy people and people attending Chinese Medicine treatment. There were 6 healthy subjects and 9 patients in the group. About 2/3 of them were male. They were divided into 3 groups conveniently and a focus group session was arranged for each group. In the cognitive debriefing session, they were asked to answer the 78 questions and then to think aloud how they understood the items and commented on the linguistic and semantic clarity of the items. They were also asked to suggest ways to improve the items. Many items were revised to make them more understandable to lay people. There were two items asking two independent things in one question. Those items were split into two items. There were another two items found written quite badly, and were not able to be improved. Therefore, they were removed from the item pool. As a result, the total number of item in the item pool remained as 78. (Table 1)

### Demographics of the sample in the field study

A sample of 273 subjects was recruited for the field study. About 63% of the subjects were recruited from provinces in Southern China and the rest from Northern China. About half of the subjects were male. The mean age was 40 years, ranging from 18 to 68 years. Most of the subjects came from the ethnic group of Han. About 66% were married. About 42% had completed secondary or technical school, and about 29% had completed university or above. There were about 18% workers, 7% farmers, 14% students, 24% professionals. About 62% were living in city, 12% in county, 9% in town and 16% in rural areas. Of the total sample, about 33% were recruited from out-patient clinics and 37% from in-patient services of Chinese Medicine hospitals. The other 30% were healthy subjects recruited conveniently in the community. (Table 2) There were no statistically significant difference among the three groups in gender, age, marital status, education level, occupation and living area. The self-reported health status was significantly better in the healthy group as compared with the patient groups.

**Table 2:** Demographic characteristic of the sample of the field test

	**Number of subjects (%)**			
	
**Demographics**	**Healthy Subjects**	**Patient subjects**		**All Subjects**
			
		**Out-patients**	**In-patients**	
	
No. of subjects	80 (29.3%)	91 (33.3%)	102 (37.3%)	273 (100%)
Source of sampling:				
Northern China	33 (41.3%)	34 (37.4%)	34 (33.3%)	101 (37.0%)
Southern China	47 (58.8%)	57 (62.6%)	68 (66.7%)	172 (63.0%)
Gender				
Male	40 (50.0%)	46 (50.5%)	51 (50.0%)	137 (50.2%)
Female	40 (50.0%)	45 (49.5%)	51 (50.0%)	136 (49.8%)
Age (Years)				
Mean (SD)	38.8 (14.1)	39.1 (14.0)	40.6 (14.6)	39.6 (14.1)
range	18–68	18–65	19–67	18–68
Ethnic group				
Han	74 (92.5%)	84 (92.3%)	95 (93.1%)	253 (92.7%)
Hui	4 (5.0%)	6 (6.6%)	5 (4.9%)	15 (5.5%)
Others	2 (2.5%)	1 (1.1%)	2 (2.0%)	5 (1.8%)
Marital status				
Single	30 (37.5%)	26 (28.6%)	21 (20.5%)	77 (28.2%)
Married	48 (60.0%)	60 (65.9%)	71 (69.6%)	179 (65.6%)
Partnered	0 (0.0%)	1 (1.1%)	5 (4.9%)	6 (2.2%)
Separated/Divorced	1 (1.25%)	1 (1.1%)	1 (1.0%)	3 (1.1%)
Widowed	1 (1.25%)	2 (2.2%)	2 (2.0%)	5 (1.8%)
Missing	0 (0.0%)	1 (1.1%)	2 (2.0%)	3 (1.1%)
Education level				
Primary school	2 (2.5%)	6 (6.6%)	10 (9.8%)	18 (6.6%)
Middle school	9 (11.25%)	28 (30.8%)	21(20.6%)	58 (21.2%)
High school	9 (11.25%)	12 (13.2%)	26 (25.5%)	47 (17.2%)
Technical school	5 (6.25%)	9 (9.9%)	13 (12.7%)	27 (9.9%)
College diploma or degree	11 (13.75%)	15 (16.5%)	16 (15.7%)	42 (15.4%)
University degree or above	44 (55.00%)	20 (22.0%)	15 (14.7%)	79 (28.9%)
Other	0 (0.0%)	1 (1.1%)	1 (1.0%)	2 (0.7%)
Occupation:				
Worker	10 (12.5%)	18 (19.8%)	22 (21.6%)	50 (18.3%)
Farmer	2 (2.5%)	8 (8.8%)	10 (9.8%)	20 (7.3%)
Professionals	32 (40.0%)	21 (23.1%)	12 (11.8%)	65 (23.8%)
Student	19 (23.8%)	13 (14.3%)	7 (6.9%)	39 (14.3%)
Unemployed	4 (5.0%)	4 (4.4%)	14 (13.7%)	22 (8.1%)
Other	11 (13.8%)	25 (27.5%)	33 (32.4%)	69 (25.3%)
Missing	2 (2.5%)	2 (2.2%)	4 (3.9%)	8 (2.9%)
Living Arrangements:				
City	62 (77.5)	51 (56.0%)	56 (54.9%)	169 (61.9%)
County	4 (5.0%)	14 (15.4%)	14 (13.7%)	32 (11.7%)
Town	1 (1.3%)	10 (11.0%)	13 (12.7%)	24 (8.8%)
Rural area	13 (16.3%)	14 (15.4%)	16 (15.7%)	43 (15.8%)
Missing	0 (0.0%)	2 (2.2%)	3 (2.9%)	5 (1.8%)
Self-reported health status:				
Very poor	0 (0.0)	2 (2.2%)	12 (11.8%)	14 (5.1%)
Poor	3 (3.8%)	15 (16.5%)	26 (25.5%)	44 (16.1%)
Neither poor nor good	20 (25.0%)	40 (44.0%)	38 (37.3%)	98 (35.9%)
Good	48 (60.0%)	30 (33.0%)	22 (21.6%)	100 (36.6%)
Very good	8 (10.0%)	4 (4.4%)	4 (3.9%)	16 (5.9%)
Missing	1 (1.3%)	0 (0.0%)	0 (0.0%)	1 (0.4%)

### The final ChQOL structure

A series of exploratory factor analysis and confirmatory factor analysis were used to test the appropriateness and structure fitness of the initial ChQOL model. The first round of exploratory factor analysis was done on the 52 items under the Physical Form domain and the Spirit domain. A two-factor structure, explaining 38% of the total variance, was derived. Factor 1 consisted mostly of items under the Physical Form domain, and factor 2 consisted of items under the Spirit domain. We had excluded items with factor loading smaller than 0.4 on either factor. Items that loaded more or less the same on both factors were also excluded. There were 20 items remained under the factor on Physical Form, and 12 items under the factor on Spirit.

Further exploratory factor analysis was conducted on the two domains independently. Factor analysis of the 20 items under the Physical Form domain resulted in 5 factors. The factors corresponded to the facets on stamina, complexion, appetite and digestion, sleep, and adaptation to climate respectively. The other facets under the Physical Form domain, including body builds, breathing, sex, bowel and urination were not supported by the data. No item under the Physical Form domain was further reduced in this step.

Factor analysis on the 12 items in the Spirit domain supported all the 4 facets in the domain. No item was reduced from the domain in this step.

Exploratory factor analysis on the 26 items under the Emotions domain resulted in four factors. The date supported the facet on joy and anger. Items under facets on worry, pensiveness and grief combined to form a factor that could be summarized as depressed mood. Some items under anxiety were included in the facet on fear. As a result, 8 items were removed and 18 items remained in the four facets, i.e. joy, anger, depression mood, and fear.

### Item-facet and facet-domain structure fitness

A series of confirmatory factor analysis on item-facet fitness were done. Nearly all the item-facet structures were supported with Comparative Fitness Index (CFI) exceeding 0.9. We therefore concluded that all the item-facet structures of the ChQOL were supported (Table 3).

**Table 3:** Structure fitness of facets, domain and the overall structure

**CHQOL facets**	**Number of items**	**CFI Item-facet**	**Chi sq**	**Degree of freedom**
Ch1.1 Complexion	4	0.928	38.07	1
Ch1.2 Sleep	3	0.946	13.73	2
Ch1.3 Stamina	6	0.994	11.70	8
Ch1.4 Appetite & digestion	4	0.985	7.29	1
Ch1.5 Adaptation to climate	3	0.820	38.42	2

Ch2.1 Consciousness	3	0.967	10.13	2
Ch2.2 Thinking	5	0.982	20.02	4
Ch2.3 Spirit of the eyes	2	1.000	0.00	0
Ch2.4 Verbal expression	2	1.000	0.00	0

Ch3.1 Joy	4	0.990	6.97	1
Ch3.2 Anger	5	0.961	20.09	4
Ch3.3 Depressed mood	6	0.982	19.04	8
Ch3.4 Fear & Anxiety	3	0.991	3.67	2

**ChQOL domains**	**No. of facets**	**CFI Facet-domain**	**Chi sq **	**Degree of freedom**

Physical Form Domain	5	0.943	295.03	159
Vitality & Spirit Domain	4	0.956	133.99	45
Emotion Domain	4	0.872	437.36	126

Facet and domain fitness of the 3 domains were also supported by confirmatory factor analysis. The CFI of the Physical form and the Sprit domain were 0.943 and 0.956 respectively. The CFI of the facet and domain structure of the Emotion domain was 0.872. (Table 3)

### Domain-overall ChQOL structure fitness

**Figure 2:**
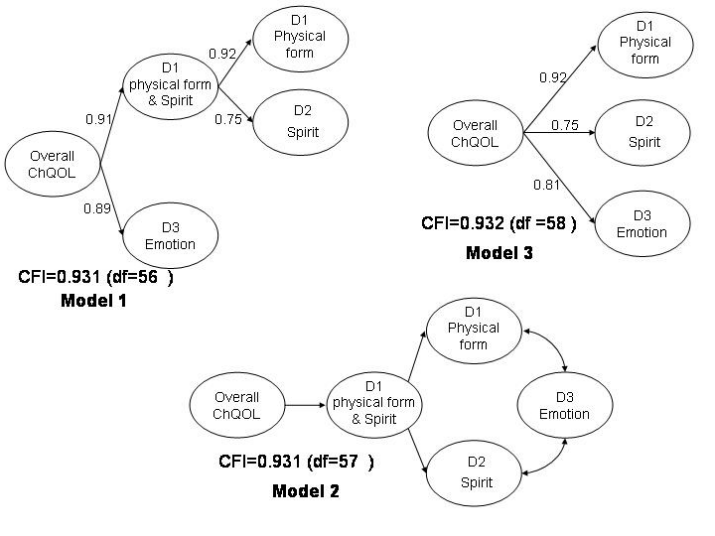
Possible final structure models of the ChQOL

**Figure 3:**
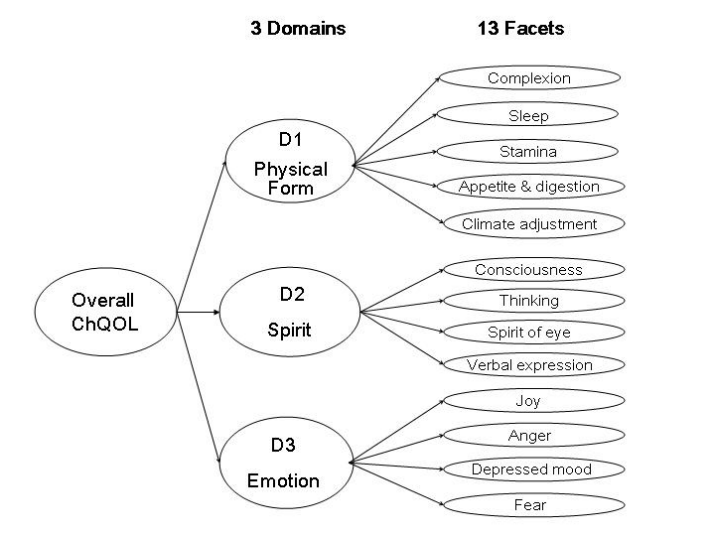
Final domains and facets structure of the ChQOL

We had proposed the 3 possible domain structures of the ChQOL (Figure 2). All of them were compatible with the concept of health in TCM. We used the domain scores to test these three possible structures. The CFI of all the three models had exceeded 0.9 and were satisfactory. Model 1 was a 3-level structure consisting of domains and sub-domains. Since the composite domain on Physical Form and Spirit did not provide much interpretable information on top of the two separate Physical form domain and Spirit domain, model 1 was aborted. Model 2 was also a 3-level structure. It was a complex model and the relationship of the domains was difficult to interpret. Hence, this model was not adopted. Model 3 is a simple 2-level model. The CFI of this model was 0.932. It was therefore adopted as the final structure. The domain and facets of the final model were shown in Figure 3. The items of the ChQOL in the original Chinese and tentative English translation were listed in Appendix 1 (Additional file 1).

### Psychometric properties of the ChQOL

#### Distribution of scores

The mean facet scores ranged from 55 in the Anger facet to 79 in the Fear facet. Mean domain scores ranged from 61 in the Physical Form domain to 69 in the Spirit domain. No ceiling and floor effects were noted. (Table 4)

**Table 4:** Distributions of the facets & domain scores (N = 273)

**ChQOL facets & Domains**	**No. of items / facets / domains**	**Mean scores (SD)**	**Floor effect (%)**	**Ceiling effect (%)**
**ChQOL facets**				
Ch1.1 Complexion	4	56.07 (18.989)	0.7	2.2
Ch1.2 Sleep	3	59.59 (22.511)	0.7	5.5
Ch1.3 Stamina	6	56.85 (19.468)	1.1	1.1
Ch1.4 Appetite & digestion	4	65.32 (18.725)	0.7	5.9
Ch1.5 Adaptation to climate	3	64.83 (19.255)	0.4	6.6
Ch2.1 Consciousness	3	71.54 (17.983)	0.4	9.6
Ch2.2 Thinking	5	64.16 (17.326)	1.1	3.3
Ch2.3 Spirit of the eyes	2	66.91 (18.508)	1.1	4.0
Ch2.4 Verbal expression	2	74.36 (18.557)	0.4	14.7
Ch3.1 Joy	4	63.26 (17.229)	0.7	2.6
Ch3.2 Anger	5	54.54 (17.788)	0.4	0.7
Ch3.3 Depressed mood	6	69.84 (18.595)	0.4	2.2
Ch3.4 Fear & Anxiety	3	79.49 (19.306)	0.4	23.4

**ChQOL domains**				
Physical form	5	60.57 (14.715)	0.4	0.4
Spirit	4	69.21 (15.745)	0.4	0.7
Emotion	4	66.78 (14.721)	0.4	0.7

**Overall ChQOL**	3	65.51 (12.764)	0.4	0.4

#### Facet to domain and inter-domain correlation

Facet to domain correlation ranged from 0.71 to 0.78 in the Physical Form domain, 0.83 to 0.91 in the Spirit domain, and 0.77 to 0.89 in the Emotion domain. The domain to overall ChQOL score correlation ranged from 0.56–0.78 (Table 5). There were moderate correlations among the domain scores. Correlation between the Physical Form and the Spirit domain, and the emotion domain were 0.56 and 0.61 respectively. Correlation between the Spirit domain and the Emotion domain was 0.54. There were high correlations between the overall ChQOL score and the three domain scores. The correlation coefficient ranged from 0.84 to 0.85.

**Table 5:** Correlation between facets and domain and overall ChQOL

**ChQOL facets**	**Pearson correlation coefficients**			
	
	**Physical Form**	**Spirit**	**Emotion**	**Overall ChQOL**
Ch1.1 Complexion	0.736**	0.553**	0.417**	0.672**
Ch1.2 Sleep	0.730**	0.264**	0.452**	0.564**
Ch1.3 Stamina	0.783**	0.534**	0.596**	0.752**
Ch1.4 Appetite & digestion	0.763**	0.400**	0.409**	0.619**
Ch1.5 Adaptation to climate	0.705**	0.366**	0.383**	0.572**

Ch2.1 Consciousness	0.518**	0.889**	0.541**	0.777**
Ch2.2 Thinking	0.498**	0.908**	0.513**	0.766**
Ch2.3 Spirit of the eyes	0.521**	0.833**	0.396**	0.700**
Ch2.4 Verbal expression	0.411**	0.846**	0.427**	0.673**

Ch3.1 Joy	0.446**	0.534**	0.774**	0.691**
Ch3.2 Anger	0.490**	0.405**	0.795**	0.662**
Ch3.3 Depressed mood	0.562**	0.434**	0.886**	0.739**
Ch3.4 Fear & Anxiety	0.468**	0.358**	0.774**	0.639**

**ChQOL Domains**				
Physical form	1.000	---	---	0.852**
Spirit	0.562**	1.000	---	0.838**
Emotion	0.609**	0.542**	1.000	0.845**

** Correlation is significant at the 0.01 level (2-tailed)

#### Reliabilities

The results on internal consistencies of the facets were all good. Cronbach's alpha values of the facets ranged from 0.71 in the Verbal Expression facet to 0.90 in the Thinking facet. Test-retest reliability study was conducted on 56 subjects within 2 days. The ICC(1,1) values ranged from 0.68 to 0.84 in the facet scores, and from 0.83 to 0.87 in the domain scores. The ICC(1,1) value for the overall ChQOL score was 0.90. (Table 6)

**Table 6:** Internal consistency and test-retest reliability of ChQOL

**ChQOL facets**	**Cronbach's alpha N = 273**	**ICC N = 56**
Ch1.1 Complexion	0.8601	0.75
Ch1.2 Sleep	0.7425	0.68
Ch1.3 Stamina	0.8484	0.82
Ch1.4 Appetite & digestion	0.8183	0.74
Ch1.5 Adaptation to climate	0.7403	0.84

Ch2.1 Consciousness	0.7949	0.67
Ch2.2 Thinking	0.9022	0.78
Ch2.3 Spirit of the eyes	0.7534	0.70
Ch2.4 Verbal expression	0.7083	0.81

Ch3.1 Joy	0.8707	0.71
Ch3.2 Anger	0.8012	0.80
Ch3.3 Depressed mood	0.8409	0.76
Ch3.4 Fear & Anxiety	0.7280	0.80

**ChQOL domains**		
Physical form	---	0.83
Spirit	---	0.87
Emotion	---	0.87
**Overall ChQOL**	---	0.90

#### Convergent Validity

Convergent and concurrent validities were studied by correlating the three domain scores of the ChQOL with the self-report health status (5-point scale ranging from very good to very bad), the six domains of the WHOQOL-100, and the eight domain scores of the SF-36.

The correlation between the three domain scores and the self reported health status were fair. The correlation coefficients were 0.56 in the Physical Form domain, 0.39 in the Spirit domain and 0.63 in the Emotion domain. All correlations were all statistically significant.

The three domain scores of the ChQOL showed fair to moderate correlation with the six domain scores of the WHOQOL-100. All the correlations were statistically significant, except the correlation between Physical Form domain in the ChQOL and Spirituality and Personal Belief domain of the WHOQOL-100. The Physical Form domain had higher correlations with the Physical domain (r = 0.73) and Level of Independence domain (r = 0.63), and had lower correlation between the Social (r = 0.31), Environmental (r = 0.36) and Spirituality domain (r = 0.08) of the WHOQOL-100. The Spirit domain had only fair correlation with all the six WHOQOL-100 domains, with correlation coefficient ranging from 0.19 in the Spirituality domain to 0.47 in the Psychological domain of the WHOQOL-100. The Emotion domain showed higher correlation with the Psychological domain (r = 0.64) and the Physical domain (r = 0.52), and lower correlation with the other 4 domains in the WHOQOL-100. The overall ChQOL score demonstrated significant correlation with all the 6 WHOQOL-100 domains. The correlation was moderate with the Physical (r = 0.64), Psychological (r = 0.63) and Level of Independence (r = 0.55) domains, and fair with the Social (r = 0.41) and the Environmental (r = 0.43) domain, and low with the Spirituality and Personal Belief domain (r = 0.21) of the WHOQOL-100. (Table 7)

**Table 7:** Correlation between ChQOL domains and WHOQOL-100 domains

**ChQOL domains**	**WHOQOL-100 domains**					
	
	Physical	Psychological	Level of independent	Social	Environmental	Spirituality
Physical form	0.730**	0.481**	0.627**	0.306**	0.358**	0.084
Vitality & Spirit	0.397**	0.470**	0.399**	0.274**	0.291**	0.187**
Emotions	0.516**	0.642**	0.382**	0.462**	0.449**	0.263**

**Overall ChQOL**	0.644**	0.628**	0.554**	0.406**	0.430**	0.208**

** Correlation is significant at the 0.01 level (2-tailed)

* Correlation is significant at the 0.05 level (2-tailed)

The three domain scores of the ChQOL showed fair to moderate correlation with all the 8 facet scores of the SF-36. All the correlations were statistically significant except the correlation between the Spirit domain of the ChQOL with the Role Emotion facet in the SF-36. The correlation coefficients had ranged from 0.16 to 0.65. The overall ChQOL score revealed moderate correlation with General Health, Vitality, and Mental Health of the SF-36, yielding correlation coefficients of 0.55, 0.53 and 0.52 respectively. (Table 8)

**Table 8:** Correlation between ChQOL domains and SF-36 facets

**ChQOL domains**	**SF-36 facets**							
	
	PF	RP	BP	GH	VT	SF	RE	MH
Physical form	0.400**	0.423**	0.398*	0.610**	0.512**	0.501**	0.170**	0.377**
Vitality & Spirit	0.233**	0.237**	0.161**	0.379**	0.320**	0.251**	0.070	0.298**
Emotion	0.279**	0.317**	0.188**	0.416**	0.522**	0.408**	0.246**	0.647**

**Overall ChQOL**	0.359**	0.386**	0.294**	0.553**	0.533**	0.458**	0.192**	0.520**

** Correlation is significant at the 0.01 level (2-tailed)

* Correlation is significant at the 0.05 level (2-tailed)

PF = physical function; RP = role physical; BP = bodily pain; GH = General health; VT = vitality; SF = social function; RE = Role emotional; MH = mental health.

#### Discrimination validity

The examinations on the ability of the mean facet, domain and overall ChQOL scores in discriminating different groups of subjects with known differences in health status were studied using ANOVA tests, with gender and age adjusted, were conducted. The total sample was categorized in groups by three different ways. Firstly, the sample was categorized as healthy subjects, out-patients and in-patients groups. This category showed statistically significant differences in the mean scores in 19 out of 13 facets, and in the Physical Form domain, the Emotion domain, and the overall ChQOL scores among these three groups. (Table 9)

**Table 9:** Differences between facet and domain scores in healthy and patient adjusted for gender and age

	**Mean facet / domain scores**							
	
**ChQOL facets**	**Healthy subjects N = 80**		**Out-patients N = 91**		**In-patients N = 102**		**F**	**P-value**^1^
			
	**Mean**	**SD**	**Mean**	**SD**	**Mean**	**SD**		
Ch1.1 Complexion	61.67	15.58	56.52	17.92	51.27	21.14	6.791	0.001** ^5^
Ch1.2 Sleep	67.29	19.88	58.24	22.00	54.74	23.50	7.338	0.001** ^3^
Ch1.3 Stamina	65.79	15.84	57.48	16.90	49.26	21.17	17.655	0.000** ^2^
Ch1.4 Appetite & digestion	69.53	17.38	67.79	18.23	59.80	19.02	7.223	0.001** ^4^
Ch1.5 Adaptation to climate	67.93	17.30	65.84	17.83	61.52	21.46	2.263	0.106

Ch2.1 Consciousness	73.33	16.31	72.80	17.52	68.98	19.48	1.488	0.228
Ch2.2 Thinking	67.81	14.54	64.34	17.23	61.13	18.94	3.162	0.044* ^5^
Ch2.3 Spirit of the eyes	70.89	15.07	68.54	16.70	62.38	21.43	4.998	0.007** ^5^
Ch2.4 Verbal expression	76.58	15.93	74.86	18.68	72.18	20.20	1.152	0.317

Ch3.1 Joy	66.72	15.75	63.80	17.44	60.05	17.72	3.372	0.036* ^5^
Ch3.2 Anger	59.44	17.95	54.18	17.44	51.03	17.25	5.197	0.006** ^5^
Ch3.3 Depressed mood	73.02	17.69	72.07	17.06	65.36	19.86	5.355	0.005** ^4^
Ch3.4 Fear & Anxiety	82.92	17.78	80.31	19.02	76.06	20.30	3.143	0.045* ^5^

**ChQOL domains**								
Physical form	66.64	12.54	61.18	13.04	55.32	15.85	14.087	0.000** ^2^
Spirit	72.02	12.97	70.14	15.00	66.20	17.85	2.954	0.054
Emotion	70.52	14.52	67.59	14.30	63.13	14.90	6.269	0.002** ^5^

**Overall ChQOL**	69.84	11.26	66.30	11.73	61.50	13.61	9.781	0.000** ^4^

**significant at the 0.01 level (2-tailed)

* significant at the 0.05 level (2-tailed)

^1^Scheffe and Dunnett T3 tests are used for post-hoc analysis.

^2^The difference is significant for the group (1,2), (1,3) and (2,3).

^3^The difference is significant for the group (1,2), (1,3).

^4^The difference is significant for the group (1,3), (2,3).

^5^The difference is significant for the group (1,3).

Secondly, the sample was categorized into another three groups according to their self-report health status, i.e. very poor and poor health, neither poor nor good health, and good and very good health. There were statistically significant differences in all the 13 facet scores, all the 3 domain scores and the overall ChQOL scores among these three groups (Table 10).

**Table 10:** Differences between facet and domain scores in subjects of different self reported health status adjusted for gender and age

	**Mean & SD of facet / domain scores**							
			
**ChQOL Facets**	**Poor & Very poor (N = 58)**		**Neither poor nor good (N = 98)**		**Good & Very good (N = 116)**		**F**	**P-value**
			
	**Mean**	**SD**	**Mean**	**SD**	**Mean**	**SD**		
Ch1.1 Complexion	43.14	21.75	54.17	16.74	64.13	15.13	30.767	0.000** ^2^
Ch1.2 Sleep	46.26	23.58	58.50	19.48	66.95	21.28	19.272	0.000** ^2^
Ch1.3 Stamina	37.00	19.78	56.55	13.55	66.83	15.62	68.083	0.000** ^2^
Ch1.4 Appetite & digestion	52.05	20.73	66.20	15.12	70.96	17.18	24.416	0.000** ^3^
Ch1.5 Adaptation to climate	57.33	21.86	62.16	18.07	70.80	17.11	10.824	0.000** ^4^

Ch2.1 Consciousness	61.64	21.34	69.76	17.24	77.80	13.90	18.749	0.000** ^2^
Ch2.2 Thinking	52.84	22.07	62.04	12.96	71.51	14.25	28.468	0.000** ^2^
Ch2.3 Spirit of the eyes	57.11	24.01	65.56	15.34	72.83	15.38	15.500	0.000** ^4^
Ch2.4 Verbal expression	66.16	22.58	73.32	17.74	79.31	15.38	9.960	0.000** ^4^

Ch3.1 Joy	55.17	19.80	61.54	15.45	68.64	15.51	13.609	0.000** ^4^
Ch3.2 Anger	46.47	18.66	54.18	15.79	58.88	17.71	10.692	0.000** ^3^
Ch3.3 Depressed mood	59.55	21.46	69.94	18.87	74.71	14.42	16.907	0.000** ^3^
Ch3.4 Fear & Anxiety	72.27	24.55	79.17	18.00	83.26	16.36	8.310	0.002** ^5^

**ChQOL domains**								
Physical form	47.16	16.00	59.52	10.74	68.08	11.69	57.891	0.000** ^2^
Spirit	59.44	20.44	67.60	12.94	75.37	12.08	23.723	0.000** ^2^
Emotion	58.37	16.83	66.21	13.74	71.37	12.43	19.210	0.000** ^2^

**Overall ChQOL**	54.99	14.92	64.46	9.55	71.63	10.01	46.131	0.000** ^2^

** Significant at the 0.01 level (2-tailed)

* Significant at the 0.05 level (2-tailed)

^1^Scheffe and Dunnett T3 tests are used for post-hoc analysis.

^2^The difference is significant for the group (1,2), (1,3) and (2,3).

^3^The difference is significant for the group (1,2), (1,3).

^4^The difference is significant for the group (1,3), (2,3).

^5^The difference is significant for the group (1,3).

Thirdly, the sample was categorized according to their self-report disease conditions, i.e. healthy, having stable chronic illness and having acute illness that required active treatment. There were statistically significant differences in 10 out of the 13 facet scores, the Physical Form domain, the Emotion domain, and the overall ChQOL score. (Table 11)

**Table 11:** Differences between facet and domain scores in subjects of different disease conditions adjusted for gender and age

	**Mean & SD of facet / domain scores**							
			
**ChQOL Facets**	**Healthy (N = 76)**		**Stable chronic illness (N = 56)**		**Receiving active treatment (N = 139)**		**F**	**P-value**
			
	**Mean**	**SD**	**Mean**	**SD**	**Mean**	**SD**		
Ch1.1 Complexion	64.67	15.82	57.11	15.49	50.99	20.29	13.656	0.000** ^2^
Ch1.2 Sleep	68.09	20.52	55.51	20.86	56.35	23.13	7.696	0.000** ^3^
Ch1.3 Stamina	67.45	16.12	57.29	14.22	50.73	20.54	19.459	0.000** ^4^
Ch1.4 Appetite & digestion	71.71	17.27	65.29	16.47	61.51	19.39	7.252	0.001** ^2^
Ch1.5 Adaptation to climate	69.00	16.81	65.63	16.44	61.93	21.00	2.604	0.076

Ch2.1 Consciousness	74.34	17.04	71.21	16.10	70.08	19.11	1.209	0.300
Ch2.2 Thinking	68.55	16.73	63.04	14.42	62.23	18.43	3.090	0.047* ^2^
Ch2.3 Spirit of the eyes	71.83	16.19	66.29	13.47	64.48	20.87	3.380	0.036* ^2^
Ch2.4 Verbal expression	77.33	17.52	73.66	16.63	73.11	19.85	1.022	0.361

Ch3.1 Joy	67.43	17.33	62.61	13.82	61.24	18.15	3.153	0.044* ^2^
Ch3.2 Anger	60.79	18.04	52.14	15.34	52.23	17.92	6.849	0.001* ^3^
Ch3.3 Depressed mood	74.01	16.46	69.72	16.64	67.42	20.12	4.112	0.017* ^2^
Ch3.4 Fear & Anxiety	83.99	17.09	81.70	15.68	76.08	21.25	5.765	0.004** ^2^

**ChQOL domains**								
Physical form	68.41	12.78	60.16	10.96	56.30	15.33	17.518	0.000** ^3^
Spirit	72.89	14.23	68.66	13.24	67.48	17.16	2.432	0.090
Emotion	71.56	13.67	66.54	12.34	64.24	15.63	7.096	0.001* ^2^

**Overall ChQOL**	71.11	11.34	65.09	9.44	62.67	13.72	10.705	0.000** ^3^

** Significant at the 0.01 level (2-tailed)

* Significant at the 0.05 level (2-tailed)

^1^Scheffe and Dunnett T3 tests are used for post-hoc analysis.

^2^The difference is significant for the group (1,3).

^3^The difference is significant for the group (1,2), (1,3).

^4^The difference is significant for the group (1,2), (1,3) and (2,3).

### Conceptual overlapping with the WHOQOL-100 and SF-36

Further exploration was then conducted to examine the degree of overlapping between the ChQOL and two other well established generic HrQOL instruments, i.e. the WHOQOL-100 and the SF36. In the exploratory factor analysis on the 13 facet scores of the ChQOL and the 24 facet scores of the WHOQOL-100, seven factors were generated that explained about 66% of the total variance in the data set. (Table 12). Factor one consisted of 4 out of 5 facets in the Psychological domain, all the 3 facets in the Social Relationship domain, 2 facets in the Environment domain and the only facet in the Spirituality and Personal beliefs domain of the WHOQOL-100. This explained about 14% of the total variance of the data. Factor two consisted of all the 3 facets in the Physical domain, and all the 4 facets in the Level of Independence domain of the WHOQOL-100, and explained about 11.7% of the total variance. Factor three consisted of all the 4 facets in the Spirit domain and 1 out of 5 facets of the Physical Form domain of the ChQOL, and explained 11.3% of the variance. Factor four consisted of 6 out of 8 facets in the Environment domain of the WHOQOL-100, and explained 10.4% of the total variance. Factor five consisted of all the 4 facets in the Emotion domain in the ChQOL and the Negative feeling facet in the Psychological domain of the WHOQOL-100, and explained 8.7% of the total variance. The 2 factors that made up mostly of facets of the ChQOL, i.e. factor 3 and 5, explained about 20% of the total variance, i.e. 1/3 of the total variances that could be explained by all the seven factors.

**Table 12:** Exploratory Factor analysis on the 24 facets of WHOQOL-100 and the 13 facets of ChQOL

**Facets**	**Component**						
	
	**1**	**2**	**3**	**4**	**5**	**6**	**7**
F2.1 Positive Feelings	.663						
F2.2 Thinking, Memory & Concentration	.604						
F2.3 Self-esteem	.712						
F2.4 Bodily Image and Appearance	.521						
F4.1 Personal Relationships	.779						
F4.2 Social Support	.643						
F4.3 Sexual Activity	.607						
F5.5 Acquiring New Information & Skills	.563			.509			
F5.6 Participation in Leisure	.675						
F6.1 Spirituality/ Religion/ Personal Beliefs	.533						
F1.1 Pain & discomfort		-.604					
F1.2 Energy and Fatigue		.655					
Ch1.3 Stamina		.604					
F3.1 Mobility		.768					
F3.2 Activities of Daily Living		.723					
F3.3 Dependence on Medication		-.723					
F3.4 Working Capacity		.650					
Ch1.1 Complexion			.535				
Ch2.1 Consciousness			.807				
Ch2.2 Thinking			.869				
Ch2.3 Spirit of Eyes			.776				
Ch2.4 Verbal Expression			.786				
Ch1.5 Adaptation to climate							
F5.1 Physical Safety and Security				.547			
F5.2 Home Environment				.730			
F5.3 Financial Resources				.704			
F5.4 Availability & quality of health & social care				.676			
F5.7 Physical Environment				.711			
F5.8 Transport				.763			
Ch3.1 Joy					.476		
Ch3.2 Anger					.664		
Ch3.3 Depressed mood					.765		
Ch3.4 Fear					.681		
F2.5 Negative Feelings					-.570		
F1.3 Sleep and Rest						.776	
Ch1.2 Sleep						.745	
Ch1.4 Appetite & digestion							.580
Variance (%)	13.98	11.74	11.29	10.38	8.68	6.28	3.63
Cumulative (%)	13.98	25.72	37.01	47.40	56.08	62.37	66.00

Extraction Method: Principal Component Analysis; Eigenvalue >1; Varimax rotation with Kaiser normalization; sorted by factor names; factor loading <0.5 suppressed

Facet labels begin with F are WHOQOL-100 facets

Facet labels begin with Ch are ChQOL facets

Similar exploratory factor analysis was conducted on the 8 facets of the SF-36 and the 13 facets of the ChQOL. Four factors which explained about 64.5% of total variance of the data were identified (Table 13). Factor one consisted of 7 out of the 8 facets of the SF-36, and explained about 18.8% of the total variance. Only the facet on mental health was not included. Factor two consisted of all the 4 facets of the Spirit domain of the ChQOL, and explained about 17.8% of the total variance. Factor three consisted of all the 4 facets of the Emotion domain of the ChQOL and the Mental Health facet of the SF-36, and explained about 16.0% of the total variance. Factor four consisted of all the 5 facets of the Physical Form domain of the ChQOL, and explained 11.8% of the total variance. The three factors that consisted of facets mainly from ChQOL, i.e. factor 2, 3 and 4 could explain more than 2/3 of the total variance.

## Discussion

We have established the item-facet-domain structure of the ChQOL based on our understanding of the concept of health in TCM. An item pool consisting of 78-items was developed for field testing. The ChQOL structure was validated and the items in the ChQOL reduced to 50-items. Psychometric properties of the ChQOL were good. Construct validity was established by various methods, i.e. the internal consistency in all facets were good; the correlation between facets to domain, and domains to overall ChQOL correlation were high; confirmation factor analysis showed that the structure fitness of all facets, domains and overall structure were good. Test-retest reliability was also good, especially in the domain scores with ICC value ranging from 0.83 to 0.90. No ceiling or floor effect was noted. This indicated that ChQOL could be applied to subjects with a wide range of health status. Most facet scores, domain scores and the overall ChQOL scores were able to discriminate groups of subjects with known differences in health status. The ChQOL had mild positive convergence with the other generic health related QOL measures, i.e. the WHOQOL-100 and the SF-36, with moderate correlations. This suggested that the ChQOL was measuring similar but not the same concept as compared with the two measures.

The findings showed that the ChQOL encompasses several aspects of health that are not well covered by the two existing generic HRQOL measures, i.e. the WHOQOL-100 and SF-36. Some of the examples are: the facets on complexion, appetite and digestion, adaptation to climate, spirit of eyes, and verbal expression. The ChQOL categorizes physical aspect of health into two domains, the Physical Form domain and the Spirit domain. The ChQOL contains a domain on emotion that covers various kinds of moods. This domain captures information that is affective in nature. Other psychological mechanisms, for example, self-esteem, that are more cognitive in nature are however not included.

Results indicated that there was only mild overlapping in the WHOQOL-100 and the ChQOL at the domain level, i.e. the WHOQOL-100 domains did not cover the important facets in the ChQOL and vice versa. ChQOL did contribute additional information on top of the WHOQOL-100. There were even less overlapping between SF-36 and ChQOL and both together could provide a wider range of information on people's health.

In the process of drafting items for the ChQOL, we decided to exclude the domain on Harmonization of Man and Nature, and the Harmonization of Man and Society from the ChQOL, because we were not able to draft items basing on how CM practitioners ask questions on these two domains. Since the social domain and environmental domain of life are two essential components in QOL, we may explore ways of developing these two domains to further widen the coverage of the ChQOL. To develop domain on social and environment, we may have to explore into the traditional and contemporary Chinese culture rather then Chinese medicine.

Since the ChQOL consists of 50 items and requires at least 10 to 20 minutes for a person to response to all the questions. We will explore the need and feasibility of developing a shorter version of the ChQOL for screening purpose.

We speculate that the ChQOL is more sensitive to health changes brought about by Chinese Medicine. We are planning for the next phase of study on testing the sensitivity of ChQOL in the detection of health changes attained by Chinese Medicine.

## Limitations

We attempted to develop an instrument based on the concept of health in TCM. However, due to many methodological limitations, the structure of the ChQOL was not able to fully reflect the concept of health in TCM. The ChQOL was establish in form of a questionnaire and this could only capture information, i.e. signs, symptom, emotions and feelings, that could be consciously aware by the subjects. There might be some other important information, e.g. the pulse, which could only be assessed by CM practitioners not included in the CHQOL.

The items in the ChQOL came mainly from the conversation between CM practitioners and their patients. These questions relate mostly to diseases state rather to healthy state. This might make the items in the ChQOL biased toward disease rather than health.

We were trying to use methods that were commonly used in developing QOL measures in Western medicine in the development of the ChQOL. A linear overall-domain-facet-item structure was assumed. However, this might not be a good and sufficient representation of the concept of equilibrium of Ying and Yang in Chinese medicine. The ChQOL was reflecting the concept of equilibrium of Yin and yang in health at the item level, i.e. the rating on a particular item is reflecting the equilibrium of the corresponding facet and domain, but not at the facet or domain level. We may further explore ways to manifest the concept of equilibrium at the facet and domain level. New statistical methods and new ways of conceptualizing and developing QOL instrument may be needed to achieve this.

We relied very much on the use of exploratory and confirmatory factor analysis in establishing and testing of the structure of the ChQOL. However, the sample size was relatively small to ensure a robust structure. Further studies on the construct validity of the ChQOL are necessary.

## Conclusion

We have developed a new generic, self-reported health status instrument based on a well established theory on health in Chinese Medicine. This is one of the very few instruments which were developed based on a clear and explicit theory of health. Field test results indicated that the structure of the ChQOL was valid and the psychometric properties were good. It could provide additional information of health on top of some other generic health related QOL instruments. It is ready for use in clinical trial especially in studies related to the use of Chinese Medicine.

## Authors' contributions

Leung was the principal investigator of the study. He assumed overall responsibility of coordination and the design of the study, building the structure of the ChQOL, drafting items, training of interviewers, data analysis and interpretation, and drafting of this article. Liu contributed in the formulation of the concept of health in Chinese Medicine, drafting of items, coordinate data collection and interpretation of field test results. Zhao helped in literature review, development of the structure of ChQOL, drafting of items, and the implementation of the study. She was authorized to use part of her work in this study in her PhD thesis. Fang gave advice on the design of the study, data analysis and interpretation of the results. Chan helped in supervising the work of Zhao, building the structure of the ChQOL, and interpretation of results. Lin contributed in formulating the concept of health and helped in data collection.

**Table 13:** Factor analysis of the 8 facets of SF-36 and the 13 facets of ChQOL

**Facets**	**Component**			
	
	**1**	**2**	**3**	**4**
SF-RP Role Physical	.824			
SF-SF Social Functioning	.798			
SF-BP Bodily Pain	.702			
SF-VT Vitality	.643			
SF-GH General Health	.614			
SF-PF Physical Functioning	.604			
SF-RE Role Emotional	.592			
Ch2.1 Consciousness		.836		
Ch2.2 Thinking		.857		
Ch2.3 Spirit of Eyes		.802		
Ch2.4 Verbal Expression		.800		
Ch3.1 Joy			.657	
Ch3.2 Anger			.664	
Ch3.3 Depressed mood			.786	
Ch3.4 Fear			.622	
SF- Mental Health			.785	
Ch1.1 Complexion		.527		.488
Ch1.2 Sleep				.677
Ch1.3 Stamina				.481
Ch1.4 Appetite& digestion				.711
Ch1.5 Adaptation to climate				.559
Variance (%)	18.86	17.79	15.97	11.82
Cumulative (%)	18.86	36.66	52.63	64.45

Extraction Method: Principal Component Analysis; Eigenvalue >1; Varimax rotation with Kaiser normalization; sorted by facet names; factor loading <0.5 suppressed.

Facet labels begin with SF are SF-36 domains

Facet labels begin with Ch are ChQOL facets

## Supplementary Material

Additional File 1 Appendix 1. The items of the ChQOL in their original Chinese and tentative English translation Click here for file

## References

[B1] Chan K, Lee H (2002). The way forward for Chinese medicine.

[B2] Beijing University of Traditional Chinese Medicine (1998). Basic theories of Traditional Chinese Medicine.

[B3] Beijing University of Traditional Chinese Medicine (1998). Diagnostics of Traditional Chinese Medicine.

[B4] Scientific Advisory Committee of the Medical Outcome Trust (2002). Assessing health status and quality-of-life instruments: Attributes and review criteria. Quality of Life Research.

[B5] WHOQOL Study Protocol Division of Mental Health.

[B6] The WHOQOL Group (1995). The World Health Organization Quality of Life Assessment (WHOQOL) : Position Paper from the World Health Organization. Soc Sci Med.

[B7] Fang JQ, ed (2000). Quality of life assessment and application.

[B8] Wang SH, Li LM, Li J (2001). The application of the SF-36. Foreign Medical Sciences, Section of Social Medicine.

[B9] Hao YT, Fang JQ, Li CX, Shi ML (1999). The World Health Organization Quality of Life Assessment (WHOQOL) Chinese version. Foreign Medical Sciences, Section of Social Medicine.

[B10] Norusis MJ (2002). SPSS 11.0 guide to data analysis Upper Saddle River.

[B11] Byrne B (1994). Structural equation modeling with EQS and EQS/Windows: basic concepts, applications, and programming / Thousand Oaks: Sage Publications.

[B12] Zhao L, Chan K (2003). The health concept in Chinese medicine. Medicine and Philosophy.

[B13] Zhao L, Liu FB, Leung KF, Fang JQ, Lin LZ, Chan K (2004). Discussion on the theory and structure model of the Chinese Quality of Life Instrument. Chinese Journal of Clinical Rehabilitation.

