# Supplementary material for: Development and validation of the Chinese Quality of Life Instrument
Source: Health Qual Life Outcomes. 2005 Apr 16;3:26. doi: 10.1186/1477-7525-3-26 (PMC1090607; doi:10.1186/1477-7525-3-26)
Supplement: Additional File 1 — Appendix 1. The items of the ChQOL in their original Chinese and tentative English translation [file 1477-7525-3-26-S1.doc]

**Appendix 1. The items of the ChQOL in their original Chinese and tentative English translation**

| **Facet codes and names** | **Item No.** | **Items in original Chinese and English translations (note1)** | **Reverse polarity of the scale (note 2)** |
| --- | --- | --- | --- |
| **Physical Form Domain** |  |  |  |
| Ch1.1 Complexion | 1 | 您覺得自己面色怎麼樣？  What do you think about the colour of your face? | No |
|  | 2 | 您覺得面色有光澤嗎？  Do you think the colour of your face bright and shiny? | No |
|  | 3 | 您覺得自己的口唇顏色怎麼樣？  What do you think about the colour of your lips? | No |
|  | 4 | 您覺得自己的口唇潤澤嗎？  Do you think your lips are moist and shiny? | No |
| Ch1.2 Sleep | 5 | 您有失眠嗎？  Do you have insomnia? | Yes |
|  | 6 | 做夢影響您的睡眠質量嗎？  Do dreams affect the quality of you sleep? | Yes |
|  | 7 | 您睡眠怎麼樣？  How is your sleep? | No |
| Ch1.3 Stamina | 8 | 您走路輕便嗎？  Do you walk lightly? | No |
|  | 9 | 您容易疲倦嗎？  Do you tire easily? | Yes |
|  | 10 | 您有肢體乏力嗎？  Are your body and limbs lack strength? | Yes |
|  | 11 | 您覺得精力充沛嗎？  Do you feel full of energy? | No |
|  | 12 | 您經常感到力不從心嗎？  Do you often feel not able to do what you want? | Yes |
|  | 13 | 您容易上氣不接下氣嗎？  Do you pant easily? | Yes |
| Ch1.4 Appetite & Digestion | 14 | 您覺得吃飯香嗎？  Do you feel your food tasty? | No |
|  | 15 | 您感覺有消化不良嗎？  Do you feel indigestion? | Yes |
|  | 16 | 您的飯量正常嗎？  Is the quantity of your diet normal? | No |
|  | 17 | 您的胃口怎麼樣？  How is your appetite? | No |
| Ch1.5 Climate Adaptation | 18 | 您對季節氣候的變化能夠適應嗎？  Are you able to adapt to changes in seasons and weather? | No |
|  | 19 | 季節氣候變化對您的身體有影響嗎？  Do the changes in seasons and weather affect your body? | Yes |
|  | 20 | 早晚時辰變化對您的身體有不良影響嗎？  Do the changes of time in a day cause any adverse effect in your body? | Yes |

| **Spirit Domain** |  |  |  |
| --- | --- | --- | --- |
| Ch2.1 Consciousness | 21 | 您覺得頭腦清醒嗎？  Do you have a clear mind? | No |
|  | 22 | 您對外界的變化能做出適當的反應嗎？  Are you able to react to the external world appropriately? | No |
|  | 23 | 您能集中精神嗎？  Are you able to pay attention? | No |
| Ch2.2 Thinking | 24 | 您的記憶力好嗎？  Are you memory good? | No |
|  | 25 | 您反應快嗎？  Are your responses quick? | No |
|  | 26 | 您考慮問題思路清楚嗎？  Is your thinking clear when considering a problem? | No |
|  | 27 | 您能集中注意力思考嗎？  Are you able to concentrate on your thinking? | No |
|  | 28 | 您思維敏捷嗎？  Is your thought quick? | No |
| Ch2.3 Spirit of the Eye | 29 | 您目光有神嗎？  Do you have spirit in your eyes? | No |
|  | 30 | 您眼睛轉動靈活嗎？  Do your eyes move lively? | No |
| Ch2.4 Verbal Expression | 31 | 您說話語音清晰嗎？  Do you speak clearly? | No |
|  | 32 | 您能用語言表達自己的想法嗎？  Are you able to express your thoughts through your speech? | No |
| **Domain Emotion** |  |  |  |
| Ch3.1 Joy | 33 | 您心情愉快嗎？  Are you happy? | No |
|  | 34 | 您生活快樂嗎？  Are you living happily? | No |
|  | 35 | 您心情平靜嗎？  Do you feel peace in mind? | No |
|  | 36 | 您對生活有興趣嗎？  Are you interested in your living? | No |
| Ch3.2 Anger | 37 | 您容易心煩嗎？  Do you become annoyed easily? | Yes |
|  | 38 | 您容易發怒嗎？  Do you become angry easily? | Yes |
|  | 39 | 您容易急躁嗎？  Do you become agitated easily? | Yes |
|  | 40 | 您經常暴躁嗎？  Do you often become mad? | Yes |
|  | 41 | 您能夠控制自己的情緒嗎？  Are you able to control your emotion? | No |

| Ch3.3 Depress | 42 | 您凡事擔心嗎？  Are you worrying about almost anything? | Yes |
| --- | --- | --- | --- |
|  | 43 | 您經常感到悲傷嗎？  Do you often feel sad? | Yes |
|  | 44 | 您經常絕望無助嗎？  Do you often feel hopeless and helpless? | Yes |
|  | 45 | 您經常悶悶不樂嗎？  Are you often unhappy? | Yes |
|  | 46 | 您經常想大哭一場嗎？  Do you often want to cry? | Yes |
|  | 47 | 您經常憂愁嗎？  Are you often sad? | Yes |
| Ch3.4 Fear & Anxiety | 48 | 您經常無緣無故恐懼嗎？  Do you often feel fear without cause? | Yes |
|  | 49 | 您經常缺乏安全感嗎？  Do you often feel unsafe? | Yes |
|  | 50 | 您容易受驚嚇嗎？  Are you being scared easily? | Yes |

Note 1. The English translations in the table are tentative for illustration only. Formal translation and linguistic validation is required in the development of a formal English version.

Note 2. Reverse polarity of the scale are need for items marked with “yes”, i.e. change score 1 to 5, 2 to 4, 3 to 3, 4 to 2 and 5 to 1.
